# Establishment and risk factor assessment of the abnormal body temperature probability prediction model (ABTP) for dairy cattle

**DOI:** 10.1038/s41598-024-65419-0

**Published:** 2024-06-24

**Authors:** Tsair-Fwu Lee, Chien-Liang Chiu, Yen-Hsien Liu, Chu-Ho Chang, Jen-Chung Shao, Shih-Sian Guo, Yi-Lun Liao, Chia-Hui Chen, Chin-Dar Tseng, Pei-Ju Chao, Shen-Hao Lee

**Affiliations:** 1 Department of Electronic Engineering, National Kaohsiung University of Science and Technology, Kaohsiung, 80778 Taiwan; 2 Medical Physics and Informatics Laboratory of Electronic Engineering, National Kaohsiung University of Science and Technology, Kaohsiung, 80778 Taiwan; 3 Department of Medical Imaging and Radiological Sciences, Kaohsiung Medical University, Kaohsiung, 80708 Taiwan; 4 PhD Program in Biomedical Engineering, Kaohsiung Medical University, Kaohsiung, 80708 Taiwan; 5 School of Dentistry, College of Dental Medicine, Kaohsiung Medical University, Kaohsiung, 80708 Taiwan; 6 Department of Radiation Oncology, Linkou Chang Gung Memorial Hospital and Chang Gung University College of Medicine, Linkou, Taiwan

**Keywords:** Dairy cattle, Least absolute shrinkage and selection operator (LASSO), Abnormal body temperature probability (ABTP), Temperature-humidity index (THI), Health care, Risk factors, Engineering, Mathematics and computing

## Abstract

The study aims to develop an abnormal body temperature probability (ABTP) model for dairy cattle, utilizing environmental and physiological data. This model is designed to enhance the management of heat stress impacts, providing an early warning system for farm managers to improve dairy cattle welfare and farm productivity in response to climate change. The study employs the Least Absolute Shrinkage and Selection Operator (LASSO) algorithm to analyze environmental and physiological data from 320 dairy cattle, identifying key factors influencing body temperature anomalies. This method supports the development of various models, including the Lyman Kutcher-Burman (LKB), Logistic, Schultheiss, and Poisson models, which are evaluated for their ability to predict abnormal body temperatures in dairy cattle effectively. The study successfully validated multiple models to predict abnormal body temperatures in dairy cattle, with a focus on the temperature-humidity index (THI) as a critical determinant. These models, including LKB, Logistic, Schultheiss, and Poisson, demonstrated high accuracy, as measured by the AUC and other performance metrics such as the Brier score and Hosmer–Lemeshow (HL) test. The results highlight the robustness of the models in capturing the nuances of heat stress impacts on dairy cattle. The research develops innovative models for managing heat stress in dairy cattle, effectively enhancing detection and intervention strategies. By integrating advanced technologies and novel predictive models, the study offers effective measures for early detection and management of abnormal body temperatures, improving cattle welfare and farm productivity in changing climatic conditions. This approach highlights the importance of using multiple models to accurately predict and address heat stress in livestock, making significant contributions to enhancing farm management practices.

## Introduction

This study focuses on the impact of climate change-induced high temperature and humidity conditions on the survival challenges of lactating dairy cows, particularly how these conditions affect milk production. It investigates the effects of heat stress in subtropical climates, such as in the Southeastern United States and Taiwan, where it persists for half a year, causing significant health stress on dairy cows and considerable economic losses to the livestock industry^1^. To facilitate early detection and management of heat stress, Jaddoa et al.’s study^2^ employed efficient and non-invasive infrared thermography to specifically monitor the temperature around a cow’s eye socket. This was done in conjunction with an automated body temperature monitoring platform developed by Guo et al.^3^. The study aims to develop an abnormal body temperature probability (ABTP) model for dairy cattle, providing a powerful tool for farm managers to protect cattle from environmental stress.

Existing methods for monitoring and managing heat stress in dairy cattle traditionally involve environmental modifications, like providing shade and ventilation, and behavioral observations, such as monitoring for signs of heat stress^4,5^. However, these methods have limitations in their accuracy and timeliness. They often rely on subjective assessments and may not detect early stages of heat stress^6–8^. In contrast, technological advancements like infrared thermography offer more precise, non-invasive, and real-time monitoring of cattle body temperature, allowing for earlier intervention^8^. These new methods represent a significant improvement in both detecting and managing heat stress, enhancing the welfare of dairy cattle and the overall efficiency of dairy farming operations.

In this study, we aim to develop a dairy cattle body temperature ABTP to assist farm managers in early identification and effective management of heat stress effects on dairy cattle due to high-temperature and high-humidity environments. This is crucial for enhancing the welfare and milk yield of dairy cattle. To achieve this, the research team utilized statistical methods such as the least absolute shrinkage and selection operator (LASSO) to analyze risk factors affecting body temperature anomalies^9,10^. LASSO is a widely used technique for prediction and variable selection, handles datasets with numerous variables by compressing and selecting the most predictive ones to enhance model accuracy. Additionally, biological growth models like Logistic, Schultheiss, and Lyman Kutcher Burman (LKB) model, which simulate growth processes and cellular characteristics^11,12^ were employed to accurately predict dairy cows’ biological responses. These models help distinguish between low and high-risk assessment intervals, offering a new scientific approach to health management under heat stress, contributing to the sustainable development of livestock farming and animal welfare.

## Methods

**Figure 1:**
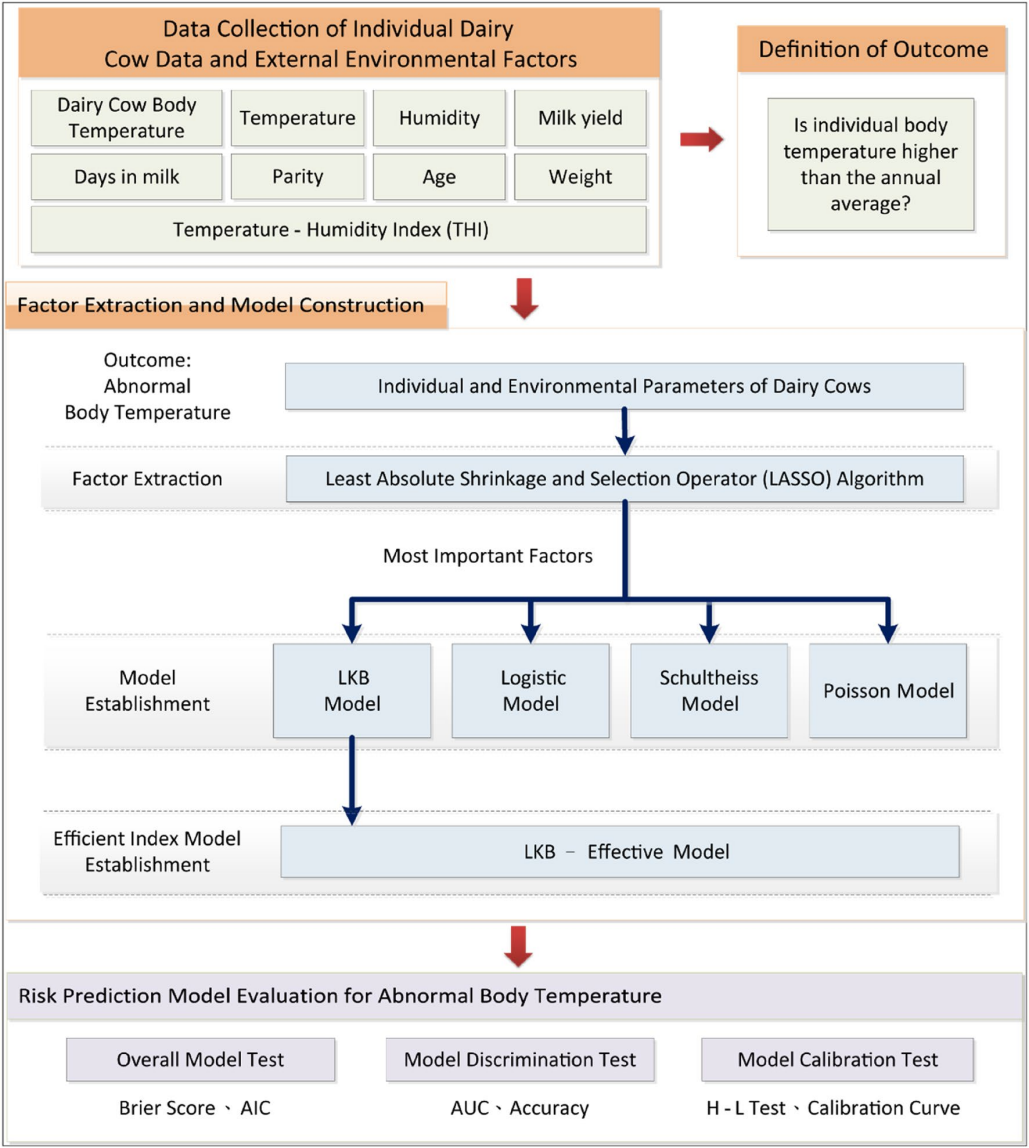
Diagram of the research process. LASSO: least absolute shrinkage and selection operator; LKB: Lyman Kutcher-Burman; AIC: Akaike’s entropic information criterion; AUC: Area Under Curve; H–L Test: Hosmer–Lemeshow test.

The study aims to develop a model capable of accurately predicting and providing early warnings for abnormal body temperatures in dairy cows. This model assists farm managers in better adapting to and addressing challenges brought by climate change. The research process is detailed in Fig. 1, with further descriptions provided below.

### Dairy cattle body temperature and environmental data analysis

From July 2019 to April 2020, we collected data from 320 Holstein–Friesian cattle at a research dairy farm operated by the Council of Agriculture’s Animal Husbandry Research Institute^13^. The dataset comprehensively included individual cattle data such as body temperature, environmental temperature, humidity, age, days in milk, parity, body weight, and milk yield, all of which are meticulously detailed in Table 1. This data was gathered using advanced tools such as the LWIR infrared thermal imager (A615 model, FLIR Systems Inc., MA, USA), known for its precision of temperature measurement within a margin of ± 2%. This instrumentation was rigorously calibrated using the SR800 blackbody furnace (CI Systems Inc, Israel) to ensure accurate temperature recordings. This methodological rigor supports the reliability of our data collection process, addressing concerns about the adequacy of our data gathering techniques.

**Table 1:** Dairy cattle individual and environmental data.

Data source	Factor (unit)	Mean ± standard deviation
Automated body temperature system	Eye socket (°C)	38.3 ± 1.40
Central weather administration, MOTC	Temperature (°C) Humidity (%) THI	25.13 ± 4.55 83.79 ± 7.90 75.37 ± 7.11
Farm database	Age (months) Days in milk (days) Parity (number) Body weight (kg) Milk yield (l)	49 ± 27 194 ± 132 2 ± 1 599.76 ± 81.07 22.27 ± 6.87

THI: Temperature-Humidity Index; MOTC: Central Weather Bureau of the Ministry of Transportation and Communications, Taiwan.

In this study, methods were conducted according to the guidelines and regulations of our country. Since the experimental design involves capturing images through surveillance without direct contact, there’s no need for approval by an institutional review board for animal participants. Moreover, all methods adhere to the ARRIVE guidelines, ensuring transparency and repeatability.

The study utilized these data to determine if an individual cow’s body temperature deviated from the annual average, indicating potential anomalies. Factor extraction and model construction were subsequently undertaken using the LASSO algorithm to identify critical factors influencing body temperature anomalies. We developed various models, including LKB, Logistic, Schultheiss, and Poisson, to assess the risks associated with abnormal temperatures. The final stage of our research involved evaluating the predictive performance of these models using statistical measures such as Brier Score, Akaike Information Criterion (AIC), Area under the ROC curve (AUC), accuracy, Hosmer–Lemeshow (HL) test, and calibration curves. This extensive process aimed to establish an early-warning system to manage the impacts of climate change on dairy cattle health effectively.

Throughout the research process, rigorous quality control measures were implemented at each stage to ensure the reliability and accuracy of the findings^14^. During the data collection phase, temperature and humidity sensors underwent regular calibration to maintain their precision. In the factor extraction and model construction phase, the LASSO algorithm’s settings were fine-tuned to prevent overfitting. For model evaluation, cross-validation techniques were used to assess the models’ performance across various data subsets. Use a predefined threshold for performance metrics like AUC, Brier Score, and HL test to ensure consistency. This multi-layered approach to quality assurance underpins the robustness of our ABTP models.

In this study, a specific temperature-humidity index (THI) designed for dairy cows was utilized, which is a comprehensive indicator considering the effects of air temperature and humidity on heat stress. THI is crucial for assessing climate adaptability and is essential in analyzing the sensitivity of animals and humans to changes in temperature and humidity. The THI conversion formula (Eq. 1)^15^ used in the study is as follows:

1 $$THI=\left(1.8\times T+32\right)-(0.55-0.0055\times RH)\times (1.8\times T-26)$$

where *T* represents the temperature parameter in degrees Celsius (°C), and *RH* represents the humidity parameter, expressed as a percentage (%).

### Application of LASSO and ABTP for dairy cattle

LASSO is effective in handling complex scenarios with multiple variables. This method compresses regression coefficients and ranks the importance of each factor. LASSO uses constraints to reduce some coefficients to zero, thereby identifying the most critical variables. The mathematical formula (Eq. 2) for LASSO provides a detailed approach for this selection and compression process.

2 $$\text{arg}{min}_{\beta }{\Vert Y-X\beta \Vert }^{2} subject\;to \Vert \beta \Vert = \sum_{j=0}^{d}{\left|\beta \right|}^{\alpha }\le t$$

where ‘d’ represents the number of selected factors, and ‘t’ is the coefficient for the constraint term.

Sigmoid curves are primarily used to describe the relationship between quantitative parameters and symptom prediction. Four different growth models were utilized to forecast the impact of specific variable parameters on the probability of abnormal body temperature. These models are: (1) LKB Model, (2) Logistic Model, (3) Schultheiss Model, and (4) Poisson Model. The mathematical formulas for these four models are detailed in Eqs. (3)–(6).

3 $$LKB=\frac{1}{\sqrt{2\pi }}{\int }_{-\infty }^{u}{e}^{\frac{{-t}^{2}}{2}}dt, u=\frac{n-{Tn}_{50}}{m\cdot {Tn}_{50}}$$

4 $$Logistic=\frac{1}{1+{e}^{-s}}, s={\beta }_{0}+{\beta }_{1}{n}_{1}$$

5 $$Schultheiss=\frac{1}{1+{(\frac{{Tn}_{50}}{n})}^{k}}$$

6 $$Poisson={2}^{-e(2.718\cdot \gamma \left(1-\frac{n}{{Tn}_{50}}\right))}$$

where ‘n’ refers to the important factor parameters selected by LASSO, ‘Tn_50_’ is the quantitative parameter corresponding to a 50% probability of abnormal body temperature, and ‘m’ and ‘γ’ are parameters for the curve’s slope.

To optimize parameters in the dairy cattle abnormal body temperature probability prediction models, Maximum Likelihood Estimation (MLE) was employed to ensure the best fit with actual data. This involved comprehensive searching and step-by-step calculations, primarily aimed at minimizing residuals by analyzing sample data. The method is based on the Log-Likelihood estimation formula (Eq. (7)).

7 $$Log-Likehood=\sum_{i=1}^{N}{y}_{i}\cdot \text{ln}\left({p}_{i}\right)+(1-{y}_{i})\cdot \text{ln}(1-{p}_{i})$$

where *yi* represents the observed data points, which can be 0 or 1, indicating the presence or absence of a characteristic or outcome. The *pi* denotes the predicted probability of the occurrence of the characteristic or outcome as predicted by the model for each data point *i*. This formula is used to measure the fit of the model to the observed data.

### ***Effective index model (LKB***_***eff***_***) for abnormal body temperature***

The LKB_eff_ model was originally proposed by Lyman et al. in 1985 for the field of radiation therapy, centers around a 50% probability threshold. It integrates radiation dosage calculations per unit volume and quantifies tolerance dosages for three types of complications^11^. In our study, we applied this effective index model to investigate the correlation between dairy cattle’s abnormal body temperature and key factors selected using the LASSO method. A curve response formula was utilized to determine the critical parameters causing a 50% probability of abnormal body temperature in the dataset. The detailed formula can be found in Eq. (8).

8 $${Tn}_{50}\left(temp\right)={Tn}_{50} (1)\cdot {temp}^{-c}$$

where ‘temp’ represents the dairy cattle body temperature parameter, where c > 0 indicates the relationship of change between body temperature and key factors.

In the LKB ABTP model, quantitative parameters ni are standardized, and their calculated ratios are applied to the corresponding effective temperature parameters ${Temp}_{eff}^{\left(i\right)}$. If these quantified key factors equal the standard reference value, then ${Temp}_{i}$ will be equivalent to the effective temperature parameter ${Temp}_{eff}^{\left(i\right)}$. The formula for effective temperature is detailed in Eq. (9). This approach involves normalizing each quantitative parameter ni and using these normalized values to calculate the corresponding effective temperature indices ${Temp}_{eff}^{\left(i\right)}$.

9 $${Temp}_{eff}^{\left(i\right)}={Temp}_{i}\cdot {\left(\frac{{n}_{i}}{{n}_{ref}}\right)}^{-c}$$

When applying the aforementioned method to the LKB model^16,17^, relevant variables are substituted into Eq. (3), resulting in Eq. (10).

10 $$u=\left({n}_{ref}-{Tn}_{50}\cdot {Temp}_{eff}^{-c}\right)/ \left(m\cdot {Tn}_{50}\cdot {Temp}_{eff}^{-c}\right)$$

**Figure 2:**
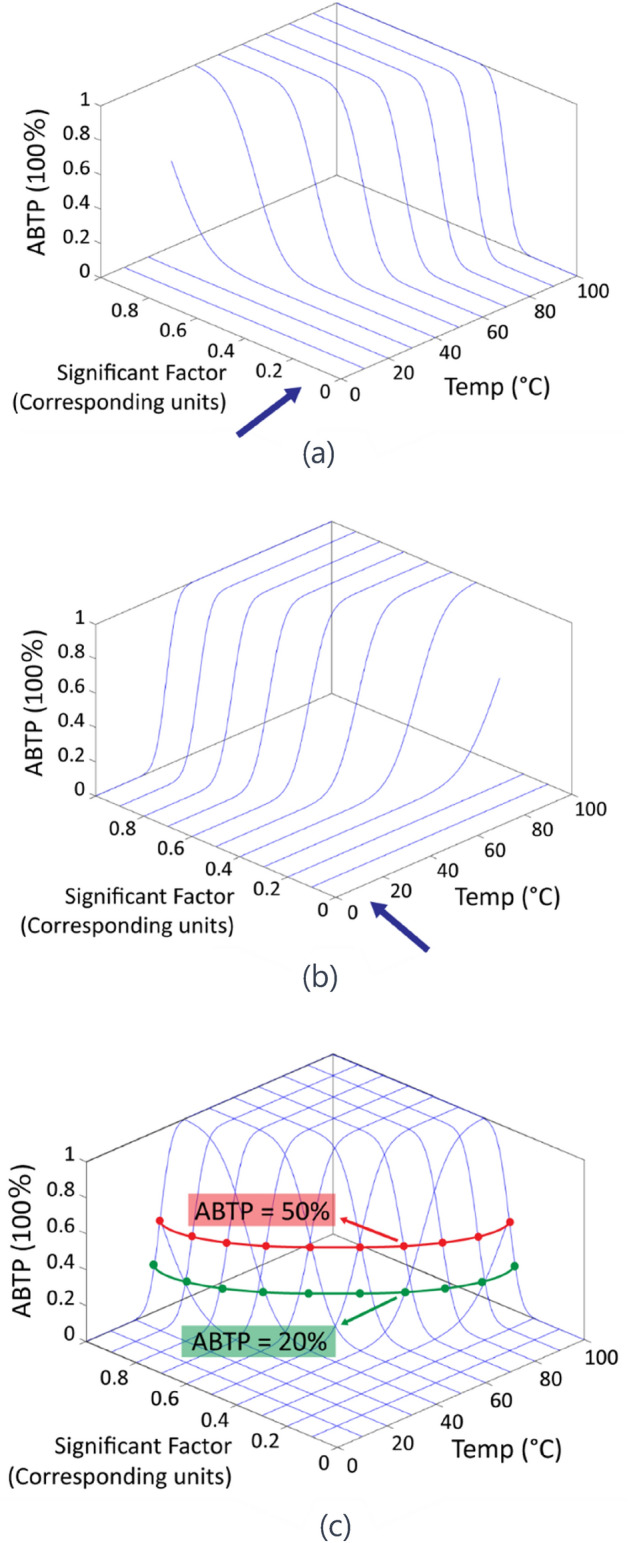
(**a**) Significant Factors from the Perspective of the ABTP Model; (**b**) Dairy Cattle Body Temperature Perspective; (**c**) ABTP_eff_ 50% and 20% Illustration. ABTP: Abnormal Body Temperature Probability, THI: Temperature-Humidity Index. The "two distinct three-dimensional models" refer to models that incorporate three dimensions of data: temperature, humidity (both contributing to the THI), and the physiological parameters of the dairy cattle like body temperature. These dimensions are crucial for evaluating the environmental and physiological factors that influence heat stress.

Figure 2 showcases the ABTP model from multiple perspectives. Part (a) identifies significant factors influencing the model. Part (b) offers insights into dairy cattle body temperature, while part (c) demonstrates the ABTP_eff_ model at 50% and 20% probability levels. This composite view provides a nuanced understanding of how various factors interplay and influence study outcomes. To illustrate this interplay, consider how temperature and humidity combine into the THI, significantly impacting dairy cattle’s heat stress levels. As THI increases, so does the risk of heat stress, which our models can quantitatively assess. For example, consider the interaction between the THI and physiological responses of dairy cattle. As THI rises, indicating higher combined effects of temperature and humidity, it leads to an increase in the cattle’s body temperature. This elevated body temperature can trigger a series of stress responses such as reduced feed intake, lowered milk production, and increased susceptibility to health issues like heat exhaustion. These responses are quantitatively analyzed using our ABTP models, which incorporate both environmental THI and physiological data to assess the risk of heat stress. The model effectively predicts the likelihood of heat stress impacts, allowing for proactive management strategies to mitigate these effects. This demonstrates the complex interactions between climatic conditions and cattle health, underscoring the relevance of our integrated modeling approach in predicting and managing heat stress.

Additionally, the study employs unique three-dimensional models (shown in Fig. 2a,b) to illustrate the data transformation process based on predefined formulas. By using the LASSO method to filter key variables, the ABTP—n_eff_ and ABTP—temp_eff_ models display curves associated with a 50% occurrence probability, forming a transverse arc curve that represents the effective temperature model trajectory. This enables rapid calculation of body temperature and key factors at different probabilities of temperature anomalies^11^.

In a subsequent phase, these 3D models are merged into a comprehensive three-factor model, depicted in Fig. 2c, which integrates multiple dimensions to enhance data analysis, focusing on displaying the 50% and 20% probability scenarios in the ABTP_eff_ model.

### Evaluation method for the dairy cattle ABTP

#### Area under the ROC curve (AUC) and accuracy

The performance evaluation of the ABTP prediction model includes testing for AUC and accuracy. The model’s predictive capability is assessed by comparing predicted probabilities with the actual occurrence of anomalies. In this study, samples with predicted probabilities exceeding 50% are classified as abnormal body temperature cases. The results allow for examining if samples conform to the model’s predictions, and the corresponding accuracy parameters are calculated^18^.

#### AIC model evaluation criterion (Akaike’s entropic information criterion)

AIC is a tool for evaluating statistical models, allowing the assessment and selection of the best model based on fundamental properties and parameter impacts. It’s calculated using log-likelihood estimates to evaluate differences between models^11,19^.

ΔAIC is computed by subtracting the AIC of the best-performing model (the one with the lowest AIC) from the AICs of the other models. In this analysis, the LKB model was used as the reference or baseline model.

#### Brier score

An indicator for evaluating the overall predictive performance of a model, where a smaller predictive error indicates better model fit^20^.

#### Calibration curve

Used to assess the consistency between actual observations and expected outcomes^21^. The ideal curve shows a 45-degree line, indicating higher accuracy of the predictive model.

#### Hosmer–Lemeshow test

Analyzes the similarity between model predictions and actual observations^22^. This test assesses the goodness of fit of a model. A result above 0.05 indicates that the model’s predicted values are not significantly different from the observed values, suggesting a good fit.

### Ethical approval and consent to participate

This study integrates non-contact methods using surveillance footage for image capture, avoiding direct interaction and thus not involving animal subjects in a manner requiring Institutional Review Board approval. All methods were conducted following national guidelines and regulations to ensure transparency and reproducibility, adhering to the ARRIVE guidelines.

## Results

### Results of factor selection using least absolute shrinkage and selection operator (LASSO)

**Figure 3:**
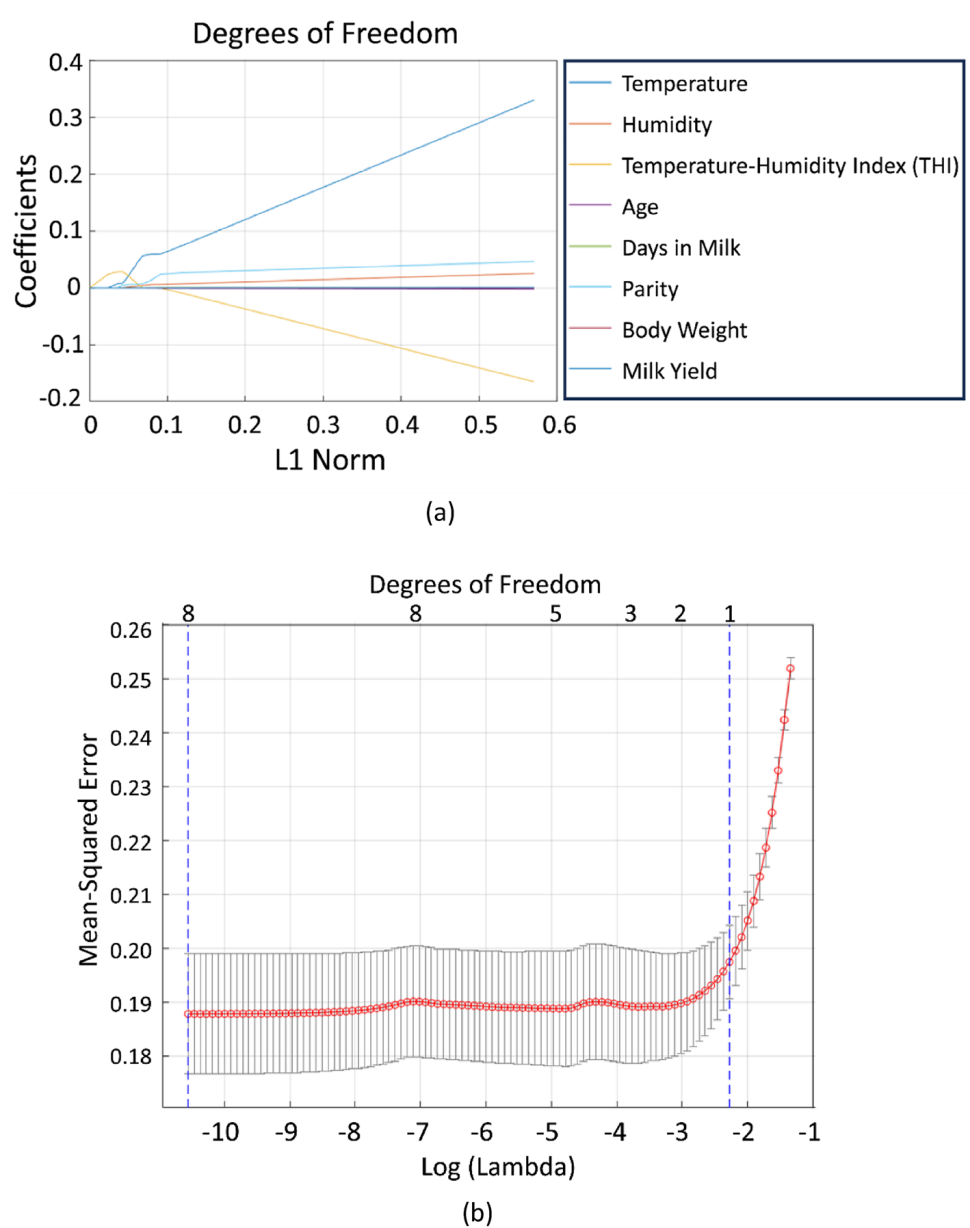
(**a**) The convergence of LASSO coefficients (**b**) the selected number of factors from the lassoglm. LASSO: least absolute shrinkage and selection operator;

In this study, LASSO was used to converge coefficients and rank them in order of importance as follows: (1) Temperature-Humidity Index (THI), (2) Temperature, (3) Parity, (4) Days in Milk, (5) Humidity, (6) Body Weight, (7) Age, and (8) Milk Yield. The convergence of LASSO coefficients is depicted in Fig. 3a, while Fig. 3b shows the number of factors selected as significant by the lassoglm method. The THI was identified as the most significant factor influencing abnormal body temperatures in dairy cattle, which was subsequently used as a quantitative parameter in the ABTP model. Analysis with the lassoglm equation confirmed THI as the key factor selected for predicting abnormal temperatures.

**Figure 4:**
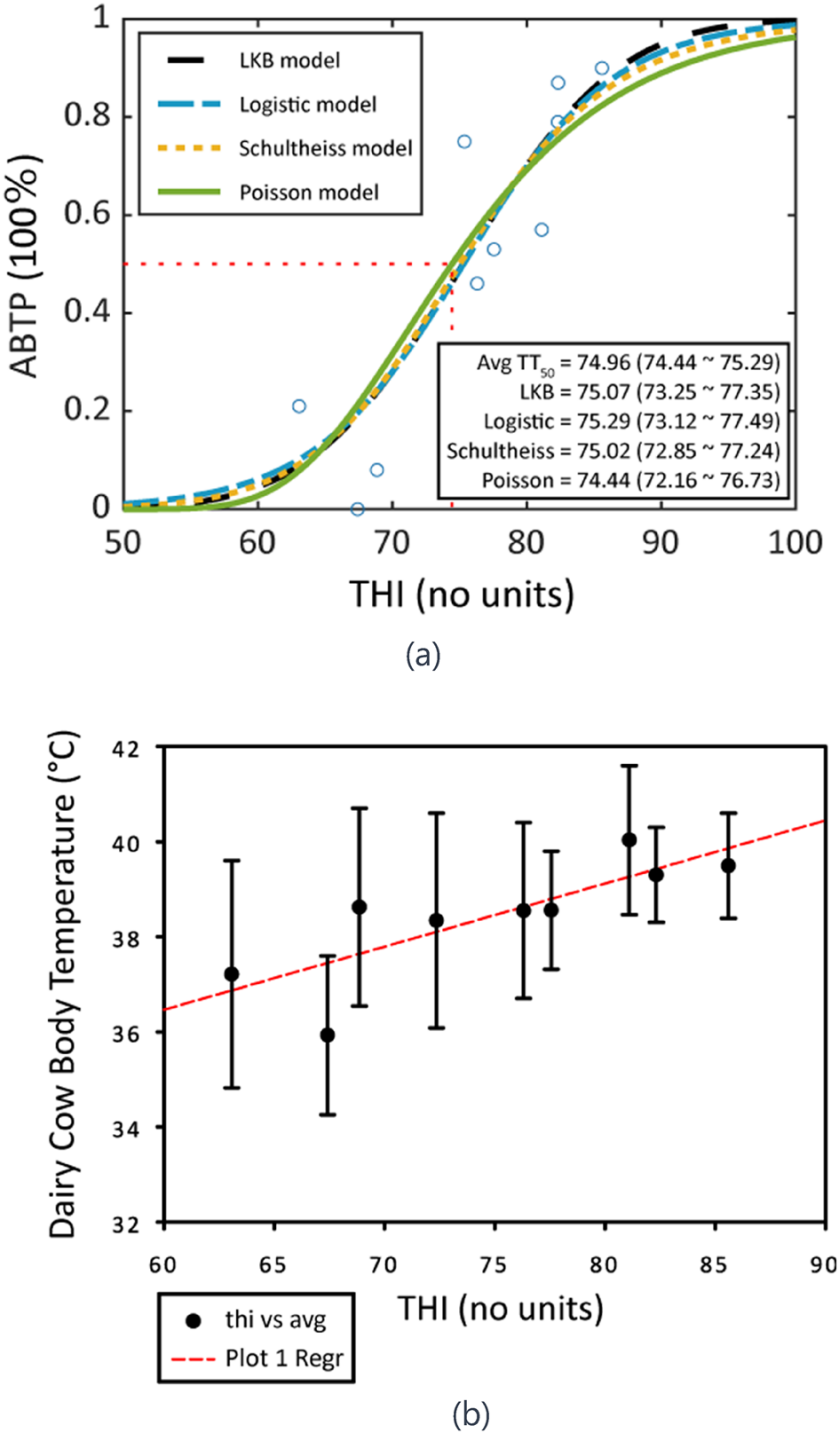
(**a**) Four Establishment of ABTP prediction models (LKB, Logistic, Schultheiss, and Poisson) (**b**) THI and dairy cattle body temperature relationship. ABTP: Abnormal body temperature probability, LKB: Lyman Kutcher-Burman, THI: Temperature-Humidity Index, $\text{THI}=\left(1.8\times \text{T}+32\right)-(0.55-0.0055\times \text{RH})\times (1.8\times \text{T}-26)$ Where T represents the temperature parameter in degrees Celsius (°C), and RH represents the humidity parameter, expressed as a percentage (%). TT_50_ represents the Temperature-Humidity Index at which there is a 50% probability of abnormal body temperature occurrence. The slope parameters for the four models are as follows: LKB: m, Logistic: γ50, Schultheiss: k, Poisson: γ.

Figure 4a illustrates the establishment and results of the LKB, Logistic, Schultheiss, and Poisson ABTP models, with TT50 values displayed at the bottom right of the graph. These visualizations provide an intuitive understanding of each model’s performance and characteristics, facilitating a comparison of their effectiveness. They lay the groundwork for further exploration and utilization of these models in research. Additionally, Fig. 4b, which plots the relationship between the THI and the body temperature of dairy cattle across 320 data entries, demonstrates that as THI increases, so does the average body temperature of the cattle. TT50 and TT20 are defined as the THI values at which there is a 50% and 20% probability, respectively, of encountering abnormal body temperatures in dairy cattle.

### Establishment and evaluation of the effective index model for abnormal body temperature

In the study, the ABTP—THI_eff_ model was crafted utilizing the LKB ABTP model’s framework to pinpoint THI values associated with 20% and 50% probabilities of abnormal body temperature predictions. Key findings indicated that a THI value of 65 corresponds with a 20% probability of ABTP, while a THI of 75 aligns with a 50% ABTP. A critical body temperature parameter of 38.32 °C was identified.

Table 2 presents the performance evaluations of four ABTP models: LKB, Logistic, Schultheiss, and Poisson. These evaluations, including predictive power, accuracy, AIC, Brier score, HL-test, and calibration curves are essential for comparing the models’ effectiveness in predicting abnormal body temperatures in dairy cattle and provide important data for further research and application. Our results suggest that all models show similar AUC, accuracy, and calibration curve values, indicating comparable predictive abilities. Variations in AIC and Brier Score may suggest differences in model fit and prediction error, while the HL-Test results across models do not show significant differences, indicating all models have an acceptable fit.

**Table 2:** ABTP model performance evaluation.

Predictive model	LKB	Logistic	Schultheiss	Poisson
AUC	0.809 (0.762–0.856)	0.809 (0.762–0.856)	0.809 (0.762–0.856)	0.807 (0.759–0.855)
Accuracy	0.713	0.713	0.713	0.706
AIC	361.28	361.60	361.56	362.22
ΔAIC	–	0.32	0.28	0.94
Brier Score	0.180	0.214	0.133	0.204
HL-Test	0.502	0.511	0.503	0.481
Calibration curve	0.897	0.897	0.896	0.891

ABTP: Abnormal body temperature probability; LKB: Lyman Kutcher-Burman; AIC: Akaike’s entropic information criterion; AUC: Area Under Curve; H–L Test: Hosmer–Lemeshow test.

**Figure 5:**
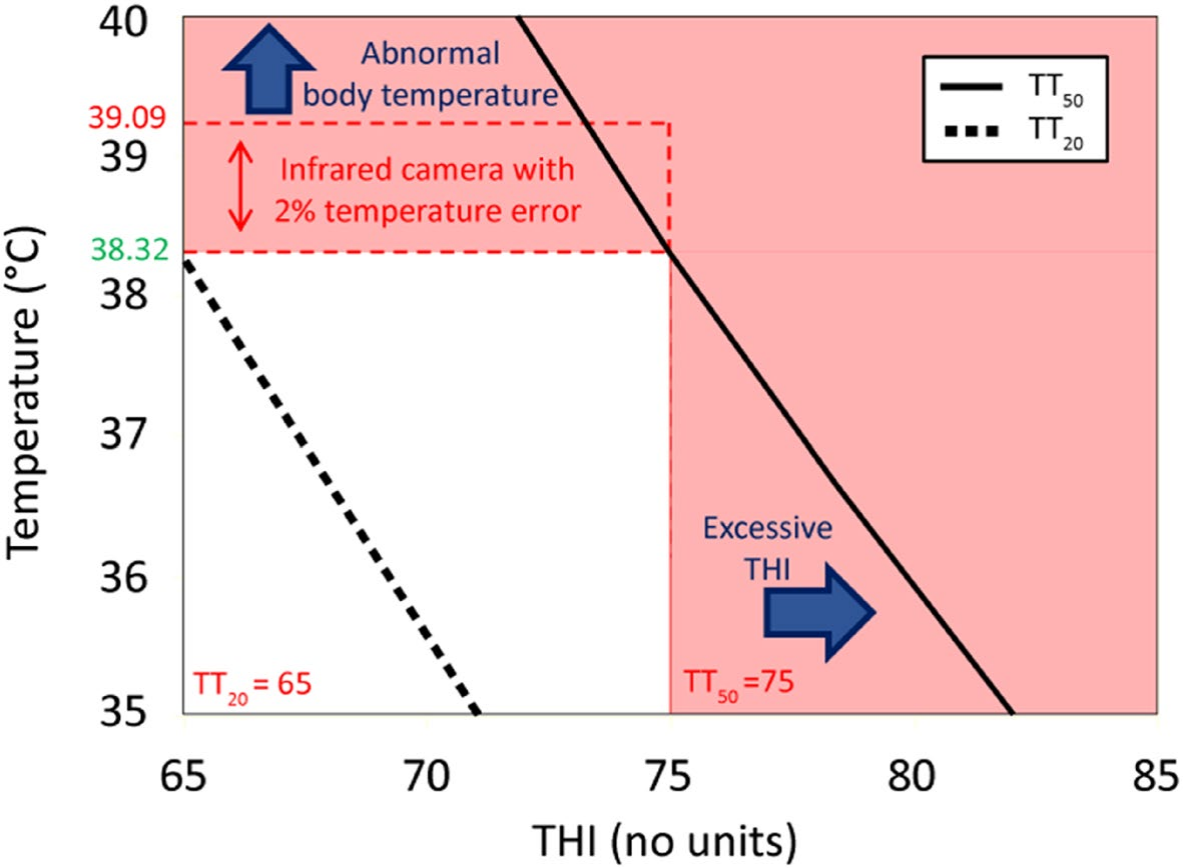
ABTP_eff_ model usage instructions. ABTP: Abnormal body temperature probability, THI: Temperature-Humidity Index. TT_50_ and TT_20_ denote the Temperature-Humidity Indices corresponding to a 50% and 20% probability, respectively, of abnormal body temperature in dairy cattle.

As Fig. 5 illustrates, the study identified an abnormal body temperature threshold for dairy cattle at 38.32 °C using the ABTP_eff_ model. Taking into account the precision and error margin of the thermal imaging device, the study defined normal body temperature as below 38.32 °C, body temperatures between 38.32 and 39.09 °C as warranting observation, and temperatures at or above 39.09 °C as abnormal, necessitating immediate notification. Furthermore, an environmental temperature-humidity alert is issued if the THI exceeds 75. The ABTP_eff_ chart is thus divided into two color blocks, with the colored area providing a reference for farm management to control cattle body temperature and THI.

## Discussion

The research presents significant findings regarding the ABTP model for dairy cattle, emphasizing the utility of the THI in predicting and managing heat stress. The study successfully integrates LASSO and biological growth models, such as LKB, Logistic, Schultheiss, and Poisson, to enhance the detection and management of heat-related risks. This provides a robust predictive framework that not only increases the accuracy of identifying heat stress but also offers actionable insights for improving farm management practices and cattle welfare.

The highlights of this study include: (1) Integration of infrared thermography and automated monitoring systems for real-time heat stress assessment in dairy cattle. (2) Application of the LASSO method to pinpoint significant predictors influencing body temperature. (3) Creation of the ABTP model, validated through statistical metrics like AUC and accuracy, to predict thermal stress effects. (4) Identification of the THI as a key environmental determinant. (5) Implementation of the ABTP model enhances farm management practices and animal welfare by providing early warning systems for heat stress management.

Our results indicate that all models demonstrate similar performance in terms of AUC, accuracy, and calibration curves, suggesting comparable predictive abilities for each. While variations in the AIC and Brier Score among the models highlight slight differences in model fit and prediction error, these differences are not substantial enough to decisively favor one model over another. Additionally, the HL Test results do not reveal significant discrepancies across the models, confirming that each has an acceptable fit for practical application. This comprehensive evaluation aids farm managers and researchers in selecting the appropriate model based on specific operational contexts, enhancing the utility of our findings in real-world scenarios where a balance between accuracy, model complexity, and computational efficiency is crucial.

A comparative analysis of the different ABTP models developed in this study. Each model—LKB, Logistic, Schultheiss, and Poisson—was designed with specific features that cater to diverse environmental and physiological variables: **LKB Model**: This model showed robust performance in environments with lower variability in temperature and humidity, which makes it particularly suitable for stable, temperate climates. Its strength lies in consistent predictions under uniform conditions^23^. **Logistic Model**: Proven to be highly effective in capturing non-linear relationships, this model excels in areas experiencing sudden changes in environmental conditions^24^. Its flexibility makes it ideal for regions with fluctuating weather patterns. **Schultheiss Model**: Best suited for regions with high humidity variations, this model offers reliable predictions in subtropical and tropical climates. Its sensitivity to humidity changes provides accurate forecasts, crucial for managing heat stress in such environments. **Poisson Model**: With its capability to handle large datasets with sparse extreme heat stress events, this model is valuable for large-scale dairy operations^25^. It ensures that predictions remain reliable even with infrequent occurrences of high heat stress levels.

The originality of this study lies in its interdisciplinary approach, combining advanced infrared thermal imaging technology, automated dairy cattle body temperature monitoring systems, and sophisticated statistical models. This comprehensive method brings innovation to the field of heat stress research. The integration of these technologies not only enhances the early detection of dairy cattle in heat stress conditions but also offers new strategies for sustainable livestock farming. In the context of global climate change, high temperatures and humidity have become major challenges in agriculture, particularly in livestock farming. These environmental changes affect animal physiology and agricultural productivity and profitability^26^. Thus, developing models that can accurately monitor and predict abnormal body temperatures in dairy cattle is crucial for improving farm management efficiency and animal welfare.

The ABTP prediction model, developed through LASSO analysis, identified key factors affecting dairy cattle body temperature, prioritizing THI^27^. This composite index of temperature and humidity renders individual factors like temperature more significant. Analyzing 320 data entries, the study confirmed THI’s correlation with body temperature. The model pinpointed the critical TT50 value, averaging 74.96, ranging from 74.44 to 75.29, indicating rising cattle body temperature with increasing THI.

Research findings similar to ours, such as those by Liu et al.^28^ and Xue^29^, emphasize the impact of the Temperature-Humidity Index (THI) on dairy cattle. These studies show that high THI levels significantly influence milk yield and physiological responses like body temperature and food intake. They also highlight that THI alone does not fully capture the physiological changes in cattle under heat stress, suggesting the need for an integrated analysis of THI with physiological parameters to better understand the dynamics of milk production.

Xue’s findings indicate a complex, non-linear relationship between THI and rectal temperature, with significant increases observed as THI rises above 72. Complementary research from Taiwan’s Council of Agriculture confirmed a consistent correlation between orbital and rectal temperatures, suggesting a sharp increase in these temperatures when THI exceeds 70, emphasizing the importance of setting appropriate THI thresholds.

Peng et al.’s research corroborates these findings, revealing a high correlation between various body temperatures and environmental THI^30^. This establishes the reliability of measuring temperatures at multiple body locations under different THI conditions, highlighting a gap in precise quantitative data linking THI to body temperature changes, thus leading to the development of the LKB THIeff model to further refine our understanding.

According to data from Taiwan’s Central Weather Bureau, temperatures in spring, summer, and autumn seasons, excluding winter, often exceed 20 °C, approaching 30 °C^31^. Using the THI index, these seasons, except for winter, can easily reach heat stress thresholds. This indicates that dairy cattle may experience varying degrees of heat stress outside of winter. The study suggests that managing environmental temperature and humidity is crucial for farm management, as prolonged standing due to heat can lead to hoof diseases and discomfort in cattle. The THI index, considering seasonal variations in temperature and humidity, serves as an effective reference throughout the year, with a THI above 75 acting as a warning indicator for potential abnormal rises in cattle body temperature.

This research incorporates "Smart Dairy Farming Technology: Comprehensive Health Monitoring of Dairy Cattle via Infrared Temperature Sensing" from the Hsinchu Branch of the Livestock Research Institute, Council of Agriculture, Executive Yuan, as a fundamental reference for confirming dairy cattle body temperatures^32^. Infrared thermal imaging tests on the farms revealed an average eye-socket temperature of 38.1 °C in cattle. Based on the ABTP_eff_ model, abnormal body temperature is defined as 38.32 °C, considering the thermal imager’s ± 2% accuracy and error margin. Normal temperatures are classified as below 38.32 °C, temperatures ranging from 38.32 to 39.09 °C require close observation, and temperatures exceeding 39.09 °C are deemed abnormal, necessitating immediate action.

In parallel, the study analyzed dairy cattle body temperature and environmental factors, confirming the THI as a crucial determinant of abnormal body temperatures. It established four predictive ABTP models—LKB, Logistic, Schultheiss, and Poisson—with an average TT_50_ value of 74.96 (ranging from 74.44 to 75.29), which corresponds to a THI of 75, marking a significant risk threshold. This THI level serves as a quantitative alert indicator for a 50% risk of abnormal body temperature. Furthermore, surpassing a THI of 75 triggers environmental heat stress warnings, advocating for proactive measures such as enhanced ventilation or cooling sprays to mitigate high temperatures’ impact and ensure cattle welfare. This integrative approach aids in establishing comprehensive early warning parameters and a predictive framework that enhances cattle management practices under varying climatic conditions.

This study on heat stress management in dairy cattle through the ABTP models provides valuable insights but has certain limitations. The primary constraints include the limited sample size of 320 Holstein–Friesian cattle from a single farm, potentially limiting the generalizability of findings across different breeds and environments. Seasonal variations and their impacts were not thoroughly analyzed, which could affect model accuracy seasonally. The reliance on the THI as a primary predictor does not capture all environmental factors influencing body temperature. Additionally, measurement errors with an accuracy margin of ± 2% and the potential for statistical overfitting or underfitting with the LASSO method could affect the precision and reliability of the findings. Practical implementation challenges such as the need for additional resources and training for effective on-farm application also pose constraints.

To address these limitations, future research could involve expanding the sample size, enhancing environmental monitoring, and testing the models under varied conditions to improve their robustness and applicability. A significant expansion plan includes conducting field tests in various tropical regions, which would allow us to confirm the models’ effectiveness across a broader range of environmental conditions. This step is crucial for their practical application in global agricultural practices, ensuring the models are adaptable and effective in different climatic challenges faced by dairy farms worldwide.

## Conclusion

This research introduces significant advancements in dairy cattle management under climate change by integrating innovative technologies and creating the novel ABTP model. The study developed multiple models, such as LKB, Logistic, Schultheiss, and Poisson, utilizing the THI to predict abnormal body temperatures and setting a critical temperature threshold of 38.32 °C. This approach not only advances scientific understanding but also provides practical tools for enhancing farm productivity and animal welfare, significantly contributing to agricultural practices and responsive health management in changing environmental conditions. Our findings offer practical solutions and methodologies that are directly applicable to improving the management of dairy cattle under changing climatic conditions, thereby filling a gap in current research and practice.

## Data Availability

The datasets used and/or analyzed during the current study available from the corresponding author on reasonable request.
